# SMYD3 contributes to a more aggressive phenotype of prostate cancer and targets Cyclin D2 through H4K20me3

**DOI:** 10.18632/oncotarget.3767

**Published:** 2015-04-25

**Authors:** Filipa Quintela Vieira, Pedro Costa-Pinheiro, Diogo Almeida-Rios, Inês Graça, Sara Monteiro-Reis, Susana Simões-Sousa, Isa Carneiro, Elsa Joana Sousa, Maria Inês Godinho, Fátima Baltazar, Rui Henrique, Carmen Jerónimo

**Affiliations:** ^1^ Cancer Biology and Epigenetics Group – Research Center, Portuguese Oncology Institute – Porto, Portugal; ^2^ School of Allied Health Sciences (ESTSP), Polytechnic of Porto, Portugal; ^3^ Life and Health Sciences Research Institute (ICVS), School of Health Sciences, University of Minho, Braga, Portugal; ^4^ ICVS/3B's - PT Government Associate Laboratory, Braga/Guimarães, Portugal; ^5^ Departments of Pathology, Portuguese Oncology Institute – Porto, Portugal; ^6^ Departments of Immunology, Portuguese Oncology Institute – Porto, Portugal; ^7^ Department of Pathology and Molecular Immunology, Institute of Biomedical Sciences Abel Salazar (ICBAS) – University of Porto, Portugal

**Keywords:** SMYD3, prostate cancer, histone methyltransferase, SET domain, cyclin D2

## Abstract

Prostate cancer (PCa) is one of the most incident cancers worldwide but clinical and pathological parameters have limited ability to discriminate between clinically significant and indolent PCa. Altered expression of histone methyltransferases and histone methylation patterns are involved in prostate carcinogenesis. SMYD3 transcript levels have prognostic value and discriminate among PCa with different clinical aggressiveness, so we decided to investigate its putative oncogenic role on PCa.

We silenced SMYD3 and assess its impact through *in vitro* (cell viability, cell cycle, apoptosis, migration, invasion assays) and *in vivo* (tumor formation, angiogenesis). We evaluated SET domain's impact in PCa cells' phenotype. Histone marks deposition on SMYD3 putative target genes was assessed by ChIP analysis.

Knockdown of SMYD3 attenuated malignant phenotype of LNCaP and PC3 cell lines. Deletions affecting the SET domain showed phenotypic impact similar to SMYD3 silencing, suggesting that tumorigenic effect is mediated through its histone methyltransferase activity. Moreover, CCND2 was identified as a putative target gene for SMYD3 transcriptional regulation, through trimethylation of H4K20.

Our results support a proto-oncogenic role for SMYD3 in prostate carcinogenesis, mainly due to its methyltransferase enzymatic activity. Thus, SMYD3 overexpression is a potential biomarker for clinically aggressive disease and an attractive therapeutic target in PCa.

## BACKGROUND

Genetic alterations have been historically considered the main driving force of cancer initiation and progression, although more recently a prominent role for epigenetic modifications has been acknowledged [1]. In addition to aberrant gene promoter methylation, alterations in chromatin modification patterns due to post-translational modifications (PTMs) of histones have been demonstrated in cancer and emerged as potential key players in neoplastic transformation. Specifically, diverse PTMs might occur in amino tail domains, including lysine acetylation, lysine and arginine methylation, serine and threonine phosphorylation, ADPribosylation, ubiquitination, and sumoylation [2]. Histone methylation is carried out by histone methyltransferases (HMTs) whereas histone demethylases (HDMs) antagonize their action [3, 4]. Depending on the target residue and the state of methylation (i.e., whether it is mono-, di- or trimethylated), PTMs may positively or negatively regulate gene transcription. In cancer cells, deregulation of HMTs or HDMs has been associated with altered post-translational control of cellular proteins affecting key signaling networks [5, 6].

Among HMTs, SET and MYND domain-containing protein 3 (SMYD3) belongs to a subfamily of SET domain-containing proteins with an important role in transcriptional regulation [7]. Its methyltransferase activity is highly dependent on two amino acid sequences, NHSC and EEL, located within the SET domain [7]. SMYD3 was firstly described as having dimethyl- and trimethyltransferase activity at lysine 4 of histone H3 (H3K4), but recently it has been reported that SMYD3 also methylates H4K5 and H4K20, as well as other non-histonic proteins, such as vascular endothelial growth factor receptor 1 (VEGFR1) [8–10]. An oncogenic role of SMYD3 has been suggested in several cancer models, including colorectal, hepatocellular, cervical and breast carcinomas [7, 11–14]. Indeed, SMYD3 was shown to induce transcriptional activation of several downstream genes, including Nkx2.8, WNT10B, RIZ1, c-Met, 15-LOX-1, MMP9 and AR [7, 11, 12, 15–18]. Interestingly, since SMYD3 is able to promote either the active H3K4me3 or the repressive H4K20me3 marks, it has been suggested that it might act either by repressing tumor suppressor genes or inducing oncogenes' expression [8].

Prostate cancer (PCa), one of the most incident cancers worldwide, remains a significant clinical challenge as PSA screening led to substantial overdiagnosis and overtreatment of patients [19]. Thus, additional efforts are needed to better identify and characterize aggressive tumors, allowing for more appropriate therapeutic strategies that will avoid unnecessary and potentially harmful interventions [20]. We have previously reported that PCa tissues displayed higher SMYD3 levels compared to normal prostate, especially at advanced disease stages. Moreover, we demonstrated that SMYD3 transcript levels convey prognostically important information and might discriminate among PCa with different clinical aggressiveness [21]. Nevertheless, how SMYD3 deregulated expression and respective histone marks impact on PCa development and progression is still largely unknown. Herein, we sought to ascertain the impact of SMYD3 methyltransferase activity on PCa cells phenotype. Knockdown of SMYD3 in LNCaP and PC3 cells attenuated the malignant phenotype, both *in vitro* and *in vivo*. This effect was mostly mediated through SMYD3 histone methyltransferase activity. Moreover, cell cycle regulation surfaced as the main cellular pathway influenced by SMYD3 methyltransferase activity, and CCND2 was identified as a putative target gene for SMYD3 transcriptional regulation through trimethylation of H4K20. Thus, a proto-oncogenic role for SMYD3 in prostate carcinogenesis is suggested, mainly due to its methyltransferase enzymatic activity.

## RESULTS

### Impact of SMYD3 silencing on the malignant phenotype of PCa cells

**Figure 1:**
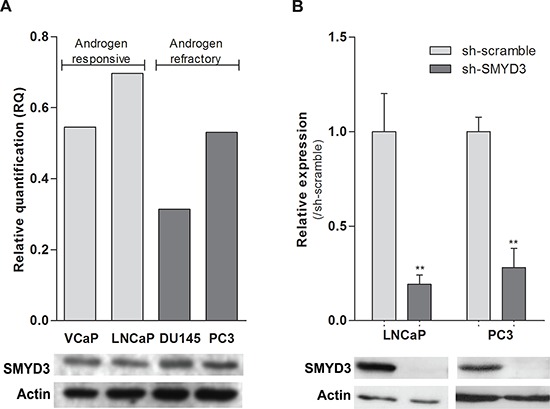
SMYD3 expression levels in PCa cell lines **A.** Expression levels of SMYD3 in VCaP, LNCaP, DU145 and PC3 cell lines. **B.** The efficiency of SMYD3 knockdown was confirmed in LNCaP and PC3 cells. mRNA levels were assessed using real-time RT-PCR (upper) and protein levels using Western-blot (lower). **p < 0.01 (Mann-Whitney *U*-test).

To select the most suitable *in vitro* model, SMYD3 expression levels were assessed in PCa cell lines LNCaP, VCaP, DU145 and PC3. All cell lines expressed SMYD3, although at variable levels (Figure 1A). The cell lines displaying the highest expression levels among the androgen-sensitive and the androgen-refractory were then selected for further analysis (LNCaP and PC3, respectively). Lentiviral particles efficiently silenced SMYD3 in those two cell lines, at transcript and protein level (Figure 1B).

**Figure 2:**
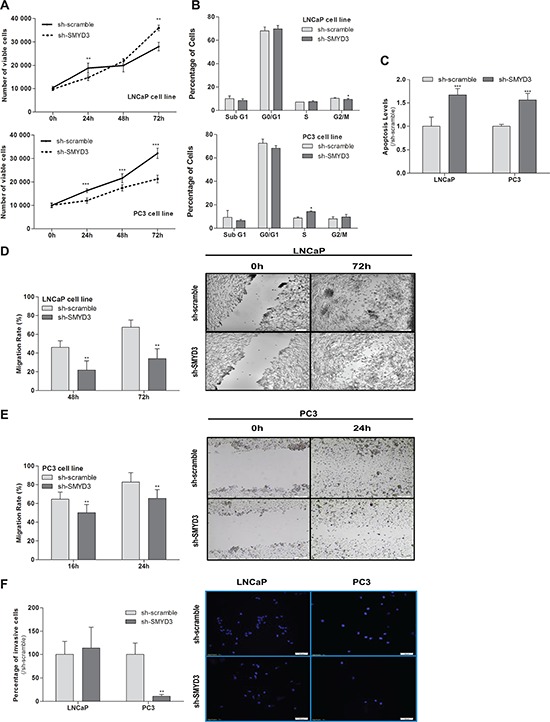
Impact of SMYD3 silencing in the malignant phenotype of PCa cells **A.** Cell viability in LNCaP and PC3: quantification of cell viability by MTT assay in sh-scramble and sh-SMYD3 at 0 h, 24 h, 48 h and 72 h in culture. **B.** Cell cycle distribution of cell lines by flow cytometry. **C.** Quantification of apoptosis by APOPercentage kit of sh-Scramble, sh-SMYD3 LNCaP and sh-SMYD3 PC3 cells at 72 h. Wound-healing scratch assay in sh-scramble and sh-SMYD3 LNCaP **D.** and PC3 **E.** cells: the left panels show the migration rate at 48 h and 72 h or 16 h and 24 h, in LNCaP and PC3, respectively, and the right panels display the illustrative images at the beginning and end point of the assay. **F.** Invasive ability was assessed by a Matrigel Invasion assay in sh-scramble and sh-SMYD3 LNCaP and PC3, cells at 48 h and 24 h, respectively. Results were normalized to the data obtained with sh-scramble cells. *p < 0.05, **p < 0.01, ***p < 0.001 (Mann-Whitney *U*-test).

The MTT assay showed a 35% decrease in viability of PC3 cells, although the opposite was apparent in LNCaP cells, at 72 h (Figure 2A). Interestingly, SMYD3-depleted PC3 cells displayed a statistically significant cell cycle arrest at S phase, whereas in LNCaP cells a reduction was depicted at G2/M transition. No significant differences in Sub-G1 population were observed for any cell line (Figure 2B). Similarly, a significant increase in BrdU uptake was observed in PC3 SMYD3-depleted cells (*p* = 0.029). Moreover, knockdown of SMYD3 induced a significant increase in apoptosis in both cell lines (Figure 2C).

A significant decrease in migration rate as well as in invasiveness capacity was also demonstrated in SMYD3-depleted PC3 cells (Figure 2E and 2F), whereas for LNCaP a statistically significant reduction was only observed in cell migration (Figure 2D).

### Effects of SMYD3 knockdown on tumor formation and vessel density *in vivo*

**Figure 3:**
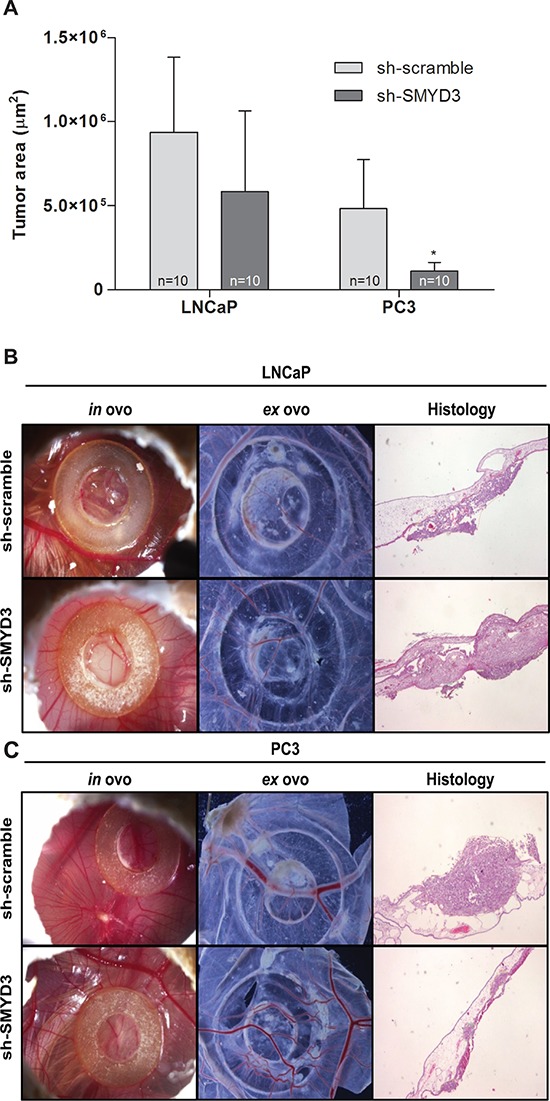
*In vivo* effect of SMYD3 silencing in tumor formation and angiogenesis in PCa cell lines **A.** Graphic depiction of tumor areas measured in histological sections. **B–C.** Representative images of CAM assay 6 days after injection of LNCaP (B) or PC3 (C) sh-scramble or sh-SMYD3 cells (*n* = 10 for each experimental condition). Images were taken *in ovo* and *ex ovo* (original magnification: × 10), as well as from histological sections (original magnification: × 40). *p < 0.05 (Mann-Whitney *U*-test).

The CAM assay was performed to evaluate the effect of SMYD3 on tumor growth and angiogenesis *in vivo* (Figure 3A–3C). The areas occupied by formed tumors were smaller in sh-SMYD3 LNCaP and PC3 compared to controls, but this difference was only statistically significant for PC3 (*p* = 0.023, Figure 3A). No statistically significant difference was apparent in linear vessel density counted *ex ovo* between sh-scramble and sh-SMYD3 cells, for both cell lines.

### SMYD3 putative oncogenic function is associated with the histone methyltransferase activity

**Figure 4:**
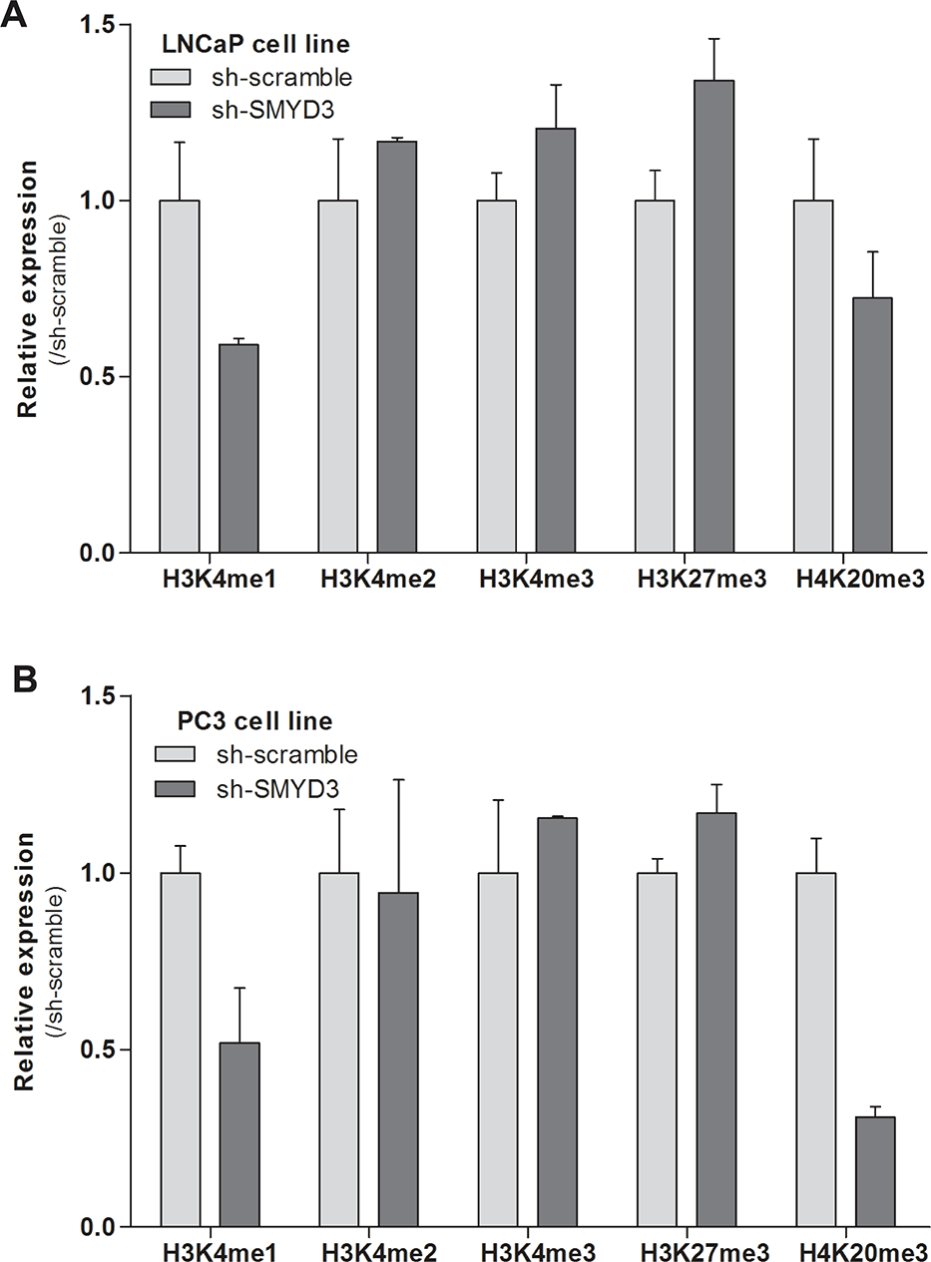
Effects of SMYD3 on global histone patterns Protein expression levels of H3K4me1, H3K4me2, H3K4me3, H3K27me3 and H4K20me3 marks in LNCaP A. and PC3 B. sh-scramble and sh-SMYD3 cells. Optical densities were normalized to histone-H3 or histone-H4 (mean ± SD, *n* = 3).

The impact of SMYD3 knockdown on mono-, di- and trimethylation levels of H3K4, as well as on trimethylation of H3K27 and H4K20 was assessed using Western-blot (Figure 4). Both LNCaP and PC3 cells showed a paradoxical increment in H3K4me2 and H3K4me3 as well as a decrease in H3K4me1, but these differences did not reach statistical significance. Interestingly, SMYD3-silenced LNCaP and PC3 cells displayed an increase (although not significant) in global levels of the repressive mark H3K27me3. Concerning the repressive mark, H4K20me3, decreased expression was displayed by both cell lines, although in PC3 the effect was more pronounced.

**Figure 5:**
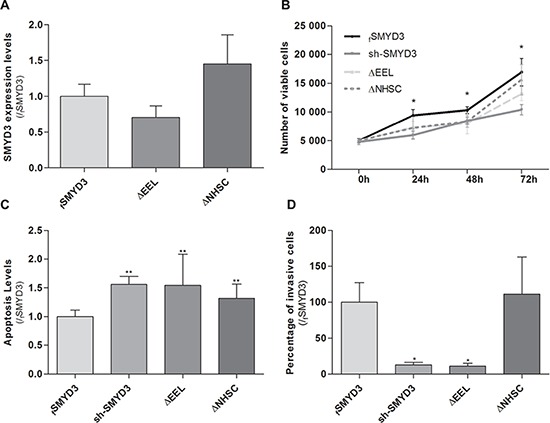
Impact of normal or mutant SMYD3 in the malignant phenotype of PC3 cells Cell viability, apoptosis levels and invasion capability were assessed in sh-SMYD3 PC3 cells and in sh-SMYD3 cells with forced expression of SMYD3 (fSMYD3) and with expression of a mutant protein with deletion of EEL (∆EEL) or NHSC (∆NHSC) region of SMYD3 SET domain. **A.** The efficiency of transfection in sh-SMYD3 was confirmed at mRNA level, using real-time RT-PCR. No significant differences in efficiency of transfection were apparent. **B.** Quantification of cell viability was performed by MTT assay after 72 h of culture. **C.** Quantification of apoptosis levels by APOPercentage kit was assessed after 72 h in culture. **D.** Invasive ability was evaluated by a Matrigel Invasion assay after 24 h of culture. Results were normalized to the data obtained with the SMYD3 normal protein (mean ± SD, *n* = 3). *p < 0.05, **p < 0.01 (Mann-Whitney *U*-test).

To assess whether the previously documented phenotypic effects were due to SMYD3 methyltransferase activity, sh-SMYD3 PCa cells were transfected with mutant SMYD3 deleted for main components of SMYD3 SET domain, the EEL and NHSC amino acid sequences and compared with forced SMYD3 expression in sh-SMYD3 PC3 cells (Figure 5A). In the absence of one of those sequences, cell viability and invasion capacity were reduced and apoptosis was increased (Figure 5B, 5C and 5D). These effects were more pronounced when the SMYD3-EEL domain was deleted. Concerning invasion capability, only SMYD3-∆EEL-PC3 cells disclosed a statistically significant reduction when compared to the control cells (Figure 5D).

### SMYD3 knockdown leads to CCND2 restored expression through downregulation of H4K20me3 mark

**Figure 6:**
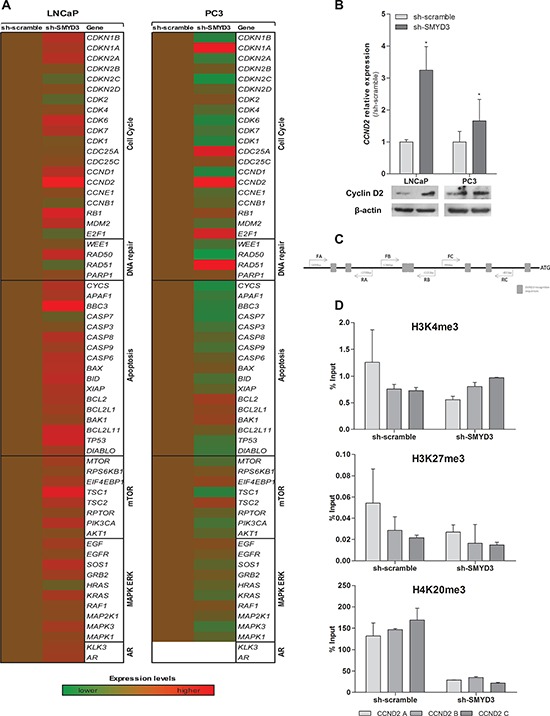
Identification of putative SMYD3 target genes and its regulation by SMYD3 histone marks **A.** Quantification of expression by custom made expression arrays of genes involved in cell cycle, apoptosis, DNA repair, mTOR or MAPK/ERK pathways normalized to sh-scramble in LNCaP or PC3 cells. **B.** Expression levels of *CCND2* using real-time RT-PCR and Western blot in both sh-scramble and sh-SMYD3 LNCaP and PC3 cell lines. **C.** Schematic representation of SMYD3 recognition sequences in Cyclin D2 promoter, used for ChIP assay. **D.** Chromatin immunoprecipitation assay for H3K4me3, H3K27me3, and H4K20me3 marks in *CCND2* promoter; primers A were located more distant upstream of Transcriptional Start-Site (−1734 bp), B (−1130 bp) and C (−840 bp) were the closest.

To identify putative target genes of SMYD3 histone methyltransferase activity, expression profile of selected genes involved in cell cycle, apoptosis, DNA repair, mTOR and MAPK/ERK pathways was evaluated after SMYD3 knockdown (Figure 6A). From the six genes that met the selection criteria, three were found to be overexpressed in SMYD3-silenced PC3 cells, and two in LNCaP. The only downregulated gene was observed in SMYD3-silenced PC3 cell line (Supplementary Table S1). Remarkably, Cyclin D2 (*CCND2*) was overexpressed both in expression array, qRT-PCR assay and western blot analysis for the two cell lines when SMYD3 expression was knockdown (Figure 6B).

**Figure 7:**
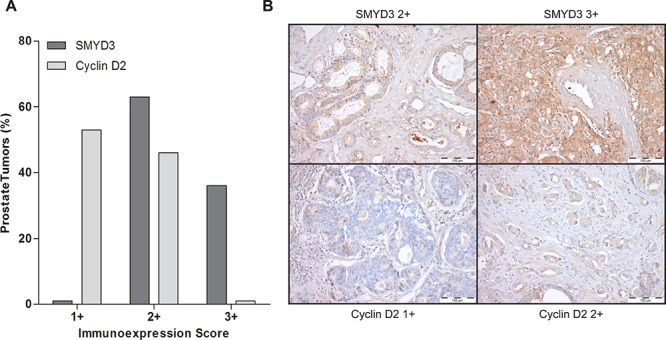
Immunoexpression of SMYD3 and Cyclin D2 in prostate cancer tissues **A.** Distribution of SMYD3 and Cyclin D2 immunoexpression in 150 primary prostate cancer tissues according to immunoscore [1+ (expression lower than in normal prostate tissue), 2+ (expression similar to normal prostate tissue), and 3+ (expression higher than in normal prostate tissue)], **B.** Illustrative images of SMYD3 and CCND2 immunoexpression in primary prostate cancer tissues.

Interestingly, in primary tumors, a large proportion of PCa cases displayed SMYD3 overexpression (immunoscore 3+), whereas cases with Cyclin D2 underexpression predominated (Figure 7). Although a trend for an inverse correlation between SMYD3 and Cyclin D2 expression is graphically suggested, no statistically significance was attained. Remarkably, when the patient cohort was stratified according to tumor grade, increased SMYD3 immunoexpression was associated with higher Gleason score (*p* = 0.002),

Moreover, ChIP analysis of CCND2 promoter region (Figure 6C) showed a decrease in the H4K20me3 mark, concomitantly with the silencing of SMYD3, although no significant differences in H3K4me3 or H3K27me3 levels were apparent in none of the three regions tested (Figure 6D).

Methylation evaluation was also performed to ascertain whether SMYD3 depletion in PC-3 and LNCaP cells led to alteration of methylation patterns at *CCND2* promoter. After SMYD3 silencing, there was no statistical difference in CCND2 promoter methylation levels in both cell lines (Supplementary Figure S1).

We also evaluated transcript levels of four SMYD3putative targets: AR, c-Met, MMP-9 and Nkx2.8 [7, 15, 18, 22]. Although no significant alterations in AR transcript expression levels were found, in both cell lines, mRNA levels of c-Met, MMP-9 and Nkx2.8 were significantly increased in silenced LNCaP cells but not in SMYD3-silenced PC3 cells, in which a decrease was observed (Supplementary Figure S2).

## DISCUSSION

PCa is one of the most prevalent cancers and a leading cause of mortality and morbidity [23]. The growing concern about overdiagnosis and consequent overtreatment of PCa patients due to PSA screening should be addressed through the identification of those cancers that are most likely to cause clinically aggressive disease. Since current clinical and pathological parameters have a limited ability to discriminate between clinically significant and insignificant PCa, emphasis should be placed on a deeper understanding of the biology of this neoplasm, which might ultimately result in the development of more efficient biomarkers. These are mandatory to improve disease management and therapeutic decision. Because histone methylation seems to play a major role in gene expression regulation, epigenetic modifying enzymes or histone modification patterns may serve as biomarkers, suitable for diagnostic, prognostic or predictive purposes in PCa patients. We have previously reported that higher expression levels of histone methyltransferase *SMYD3* associate with more advanced stage PCa and these may predict unfavorable prognosis independently of Gleason score or pathological stage. Thus, in the present study, we sought to investigate the biological role of SMYD3 and the corresponding post-transduction modifications of histones, to determine how these might impact on the malignant phenotype of PCa cells.

Because higher expression levels of *SMYD3* were found in more aggressive PCa, our strategy was based on the knockdown of this enzyme to determine its phenotypic impact in PCa cells. After achieving a stable decrease in *SMYD3* expression, cell viability, proliferation, apoptosis, migration and invasion ability assays were carried out in two PCa cell lines that are thought to largely represent *in vivo* heterogeneity of this neoplasm. Decreased expression of *SMYD3* attenuated the highly malignant phenotype of the androgen-insensitive PC3 cells, whereas, surprisingly, cell viability of the androgen-sensitive LNCaP cell line was not apparently compromised. Furthermore, *SMYD3* silencing was associated with increased cell death by apoptosis, although the underlying mechanism was not elucidated. Concerning LNCaP cells, results of cell viability and invasive capability assays contrast with those recently reported by Liu et al., but parallel their results for cell migration and apoptosis levels [18]. The discrepancies might be due to differences in methodology because the effectiveness of *SMYD3* silencing following transfection was confirmed both in our study and in that of Liu and co-workers [18]. Interestingly, the effect of *SMYD3* knockdown in LNCaP cells reported by Liu et al. mirror those we observed in PC3 cells, which are acknowledged as representing a more aggressive phenotype of PCa cells. This observation is further supported by the downregulation of c-Met, MMP-9 and Nkx2.8 genes, which are implicated in cell proliferation, invasion and migration, in SMYD3-depleted PC3. It is noteworthy that LNCaP cells display low invasion potential, a feature that may explain the opposite effect of *SMYD3* knockdown in the invasion assay of this cell line and the upregulated expression of the above mentioned genes. Both LNCaP and PC3 cells derive from PCa metastases, although in different settings: the former from a lymph node and the latter from bone. Systemic metastatic spread requires a wider spectrum of biological aggressiveness owing to the more adverse environment that cancer cells have to endure during dissemination through the bloodstream. Thus, PC3 cells display a more aggressive phenotype compared to LNCaP, implying that the baseline for each functional assay is different for each cell line. Globally, however, the results reported for *SMYD3* silencing in PCa cells are in line with those reported for hepatocellular, colorectal, cervical and breast cancers, which further supports an oncogenic role for SMYD3. In fact, functional *in vitro* studies showed that SMYD3 knockdown was associated with growth inhibition, apoptosis and reduced migration/invasion potential in cancer cell lines of those tumors [7, 11–14].

To further characterize the effect of *SMYD3* silencing in PCa cells, an *in vivo* model (the CAM assay) was conducted. A significant decrease in tumor formation was depicted in sh-SMYD3 PC3 cells, although not in LNCaP cells. The higher biological aggressiveness of PC3 cells might explain the results observed for tumor formation. However, in a mouse model, *SMYD3* knockdown was able to reduce tumor formation by LNCaP cells [18], but PC3 cells were not tested. Moreover, it has previously demonstrated that SMYD3 methylates VEGFR1 protein, increasing its kinase activity [10], and, thus, we hypothesized that SMYD3 overexpression might have an impact in new vessel formation in the *in vivo* model. However, no significant differences in linear microvessel density were apparent in both cell lines tested. Although mammalian mice models are more close to the biological conditions found in humans, the CAM assay has been used as an alternative, owing to its lower cost and less strict regulations [24, 25]. As previously mentioned by others and taking in consideration our results, CAM assay has been shown to a reliable approach compared to standard mice models for detection of alterations in tumor formation [26, 27]. Indeed, we showed different results in cell lines with distinctive aggressiveness, further supporting the results observed in the *in vitro* phenotypic assays.

Since SMYD3 establishes H4K20 di- and trimethylation and H3K4 trimethylation, the global levels of the three states of methylation of H3K4 and also H4K20me3 mark were assessed for LNCaP and PC3 cells. Furthermore, H3K27me3 levels were also assessed because it was previously hypothesized that SMYD3 could inappropriately bind to its target genes and competing with the silencing activity of repressive complexes [28]. No significant differences were apparent for any posttranslational marks in both cell lines. Interestingly, a similar global trend was observed both in SMYD3-silenced LNCaP and PC3. Indeed, slightly higher H3K27me3 and decreased H3K4me and H4K20me3 levels were found in both cell lines after SMYD3 silencing. On one hand, our findings are in line with the aforementioned hypothesis [28], although, on the other, no direct effect on the global levels of the marks directly catalyzed by SMYD3 was apparent, most probably due to the fact that there are several enzymes responsible for catalyzing the same marks. Because phenotypic effects were apparent in both tested cell lines, it is plausible that altered SMYD3 expression could only specifically affect the H3K4me status of its target genes, which might not be of sufficient magnitude to significantly alter the global H3K4 methylation levels.

In accordance with previous findings in other tumor models [7, 29], we hypothesized that the oncogenic properties of SMYD3 could depend on its histone methyltransferase activity. We tested this hypothesis by means of transfecting sh-SMYD3 cancer cells with a *SMYD3* gene mutated at the either the EEL or the NHSC amino acid sequences of the SET domain. Phenotypic assays demonstrated that the effects of a mutated SET domain largely overlap those of *SMYD3* silencing, meaning that a functional SET domain is necessary for PCa cell lines malignant phenotype. Indeed, mutant forms behave similarly to sh-SMYD3 suggesting that mutated SMYD3 without a functional SET domain was not able to increase cell viability or rescue cells from apoptosis, for both mutations (∆EEL and ∆NHSC). Although it has been reported that the two regions of the SET domain have similar roles [29], we found that the EEL domain was more closely associated with SMYD3 tumorigenic properties in PCa cells. It should be recalled, however, that previous observations were made in breast cancer cell lines and we are the first to report this effect in PCa cells. Thus, different gene sets might be affected in different tumor models and the two regions of the SET domain might have different affinity for specific gene sequences. Although this experiment strongly suggests that SMYD3 histone methyltransferase activity might be the most important for oncogenesis, the possible contribution of its enzymatic activity on cytoplasmic proteins can not be excluded.

Owing to the phenotypic effects observed due to *SMYD3* silencing, we then searched for possible target genes, focusing on some critical genes from relevant cellular pathways frequently deregulated in tumorigenesis. Surprisingly, distinct trends in gene expression were observed in LNCaP and PC3 cells, probably reflecting the different biology and malignant phenotype of these two PCa cell lines, which may explain, at the least partially, the dissimilar results of *SMYD3* silencing on the phenotype of LNCaP and PC3 cells. As stressed above, the different origins of LNCaP and PC3, which seem to determine their phenotype, are likely to be reflected at genomic and transcript levels through different patterns of activity of genes involved in critical cellular pathways.

Interestingly, one gene involved in cell cycle regulation – *CCND2* – showed a similar expression pattern in both cell lines, *i.e*., its expression was restored following SMYD3 knockdown. These results strongly suggested that *CCND2* was a putative target gene of SMYD3. Although at protein level, assessed by immunohistochemistry, no significant inverse correlation was depicted, both frequent SMYD3 overexpression and Cyclin D2 underexpression were observed in primary PCa. Interestingly, higher SMYD3 protein levels were associated with higher Gleason score suggesting that this alteration might be associated with increased tumor aggressiveness, in line with previous observations [21] and providing further support for an oncogenic role in PCa.

Both aberrant promoter methylation and histone acetylation have been implicated in the frequent CCND2 downregulation observed in PCa [30–32]. Furthermore, the *CCND2* promoter contains DNA sequences 5′-CCCTCC-3′ or 5′-GGAGGG-3′, which are specifically recognized by SMYD3 for its transcriptional regulatory functions [7]. Thus, we interrogated the histone marks in the promoter region of *CCND2* containing those motifs, using the ChIP assay. Although no significant differences were found for H3K4 or H3K27 methylation levels, a significant decrease in the H4K20me3 mark was apparent in sh-SMYD3 PCa cells.

Because the H4K20me3 repressive mark is also catalyzed by SMYD3 [8], our results indicate that SMYD3 overexpression might be also involved in *CCND2* silencing in PCa. These findings are in line with those of cell cycle analysis, in which an S phase arrest was depicted for PC3 SMYD3-depleted cells. The fact that methylation levels of *CCND2* promoter remained unaltered upon SMYD3 silencing, indicates that SMYD3′s action at *CCND2* promoter is only by is histone methyltransferse activity SMYD3. Interestingly, epigenetic deregulation of androgen receptor expression due to SMYD3 overexpression has been reported in LNCaP cells [18] and SMYD3 may also directly interact with other nuclear receptors, such as estrogen receptor [29], further reinforcing its oncogenic role. However, in our study we were not able to confirm AR as a SMYD3 target, as previously reported.

## CONCLUSIONS

In conclusion, our data provide further evidence to sustain an oncogenic role for SMYD3 in prostate carcinogenesis. In addition to its usefulness as a biomarker for clinically aggressive disease, SMYD3 overexpression might also constitute an attractive therapeutic target in PCa because its tumor-promoting properties are mostly due to its histone methyltransferase activity. Indeed, *SMYD3* silencing might be able not only to restrain the expression of proto-oncogenes but also to restore the expression of genes inadequately silenced in PCa. A more comprehensive and detailed characterization of SMYD3 target genes is, however, mandatory to fully elucidate its role in prostate tumorigenesis.

## METHODS

### Cell lines and culture conditions

Human PCa cell lines LNCaP, PC3 and VCaP were kindly provided by Prof. Ragnhild A. Lothe from the Department of Cancer Prevention at the Institute for Cancer Research, Oslo, Norway, and DU145 was offered by Prof. Fátima Baltazar from the Life and Health Sciences Research Institute at the University of Minho, Braga, Portugal. All cell lines were maintained in recommended medium, supplemented with 10% Fetal Bovine Serum (FBS; GIBCO, Invitrogen, Carlsbad, CA, USA) and 1% penicillin/streptomycin solution (GIBCO, Invitrogen), at 37°C;C and 5% CO^2^. PCa cell lines were karyotyped by G-banding (for validation purposes) and routinely tested for *Mycoplasma spp*. contamination (PCR Mycoplasma Detection Set, Clontech Laboratories Inc., Mountain View, CA, USA).

### Generation of sh-SMYD3 silenced cell lines

*SMYD3* knockdown was performed through viral transduction in LNCaP and PC3 cell lines using shRNA Lentiviral Particles (sc-61576-V; Santa Cruz Biotechnology Inc., Santa Cruz, CA) in the presence of polybrene (Santa Cruz Biotechnology Inc.) as described by the manufacturer. Additionally, control LNCaP and PC3 cells were generated using a non-target scramble shRNA (sc-108080; Santa Cruz Biotechnology Inc.). After transduction, stable clones with shRNA were selected with Puromycin dihydrochloride (cat. 631306, Clontech Laboratories Inc.) at a final concentration of 2 μg/ml or 4 μg/ml in LNCaP or PC3 cells, respectively.

### Protein extraction and western blot analysis

Cells were collected with a gum rubber-scraping device, lysed with RIPA buffer (sc-24948, Santa Cruz Biotechnology Inc.) and protein concentration was determined using BCA assay (Thermo Scientific, Waltham, MA, USA) according to manufacturer's information. Subsequently, 30 μg of total protein were separated by SDS-PAGE, transferred to nitrocellulose membranes and incubated with antibodies against anti-SMYD3 (dilution: 1:500, Abcam, Cambridge, UK), against anti-CCND2 (dilution: 1:500, Cell Signaling, Danvers, MA, USA), anti-H3K4me1 (dilution: 1:1000, Abcam), anti-H3K4me2 (dilution: 1:1000, Abcam), anti-H3K4me3 (dilution: 1:400 Abcam), anti-H3K27me3 (dilution: 1:500 Millipore, Billerica, MA, USA), anti-H4K20me3 (dilution: 1:500 Active Motif, Carlsbad, CA, USA) and as input control anti-histone H3 rabbit antibody (dilution: 1:500, Abcam), anti-histone H4 rabbit antibody (dilution: 1:500, Abcam) and β-actin (dilution: 1:8000, Sigma-Aldrich, Schnelldorf, Germany), when appropriate. The blots were developed using Immun-Star™ WesternC™ Kit according to manufacturer's indications (BioRad, Hercules, CA, USA). All the experiments were performed in triplicate. Relative optical density determination was performed using QuantityOne^®^ Software version 4.6.6. (Biorad) and proteins levels were normalized using β-actin levels as reference.

### Cell viability assay

The effect of SMYD3 on cell viability was assessed in triplicates by 3-(4, 5-dimethylthiazol-2-yl)-2, 5-diphenyltetrazolium (MTT; Sigma-Aldrich) assay. The cells were seeded in 96-well plates at a density of 1 × 10^4^ cells/well and after 24, 48 and 72 h, cells were incubated with MTT at 37°C; C for 2 h and the reaction was stopped by the addition of 100 μl/well of Dimethyl sulfoxide (DMSO) (Sigma-Aldrich) lysing for 10min. An automated plate reader (FLUOstar Omega, BMG Labtech, Offenburg, Germany) at 540 nm with a reference filter of 630nm allowed for colorimetric quantification. The absorbance value was directly proportional to the number of viable cells.

### Cell cycle analysis

Cell cycle distribution of SMYD3 knockdown and scramble PCa cells was determined by flow cytometry. Briefly, 5 × 10^5^ harvested cells were fixed overnight at 4°C;C with 70% cold ethanol. After washing with cold PBS, cells were ressuspended in staining Propidium Iodide Solution (Cytognos S.L, Salamanca, Spain) and incubated for 30 minutes at room temperature. All cells were then measured on a Cytomics FC500 flow cytometer (Beckman Coulter, Fullerton, CA, USA) and analyzed using Modfit LT (Verity Software House, Inc, Topshan, Maine, USA). Three biological replicates were performed for sh-scramble and sh-SMYD3.

### Cell proliferation analysis

Cell proliferation was evaluated using a colorimetric immunoassy (Cell Proliferation ELISA BrdU, Roche, Mannheim, Germany) at 72 hours in scramble and SMYD3-depleted PC-3 cells. Briefly, PCa cells were exposed to the pyridine analogue bromodeoxyuridine (BrdU) during 24 hours. After incorporation into DNA, BrdU was detected by immunoassay, according to manufacturer's instructions. Absorbance was measured at 370 nm in a microplate reader (FLUOstar Omega, BMG Labtech, Offenburg, Germany), subtracting the background, at 492 nm. Absorbance values directly correlate with the amount of DNA synthesis and with the number of proliferating cells. Three replicates were performed for each condition, and at least three independent experiments were carried out.

### Apoptosis assay

Apoptosis was assessed using the APOPercentage™ kit (Biocolor Ltd., Newtownabbey, Northern Ireland, UK). LNCaP and PC3 cells were seeded in the same conditions as described for MTT assay. Following an incubation period of 72 h, the APOPercentage assay was performed according to manufacturer's instructions. Quantification of apoptosis was achieved by measuring the optical density of the released dye at 550nm with a reference filter of 620nm using a FLUOstar Omega microplate reader. To normalize the OD measured in the apoptosis test to the cell number, the OD of apoptosis assay was divided by the OD of the viability assay, also performed in 96-well plates. The results of the apoptosis assay on the silenced cells were expressed as the ratio of the values obtained for scramble cells.

### Migration assay

The ability of cells of each genotype to migrate into a defect in a monolayer culture was determined using the wound healing assay. Cells were grown to full confluence in 24-well plates and scratches were performed using a 100μL tip. The medium was removed, and cells were washed with PBS and medium replaced. Scratch closure was analyzed under the microscope and images were captured at different time points. The calculations were made according to the formula [(S Time zero-S Time point/S Time zero) × 100, where S = Distance)].

### Invasion assay

Invasion capacity was analyzed through Biocoat Matrigel Invasion Chambers (BD Biosciences, Franklin Lakes, NJ, USA) according to manufacturer's protocol. Briefly, cells were placed in Matrigel inserts and allowed to migrate for 24 or 48 h at 37°C;C. Non-migrating cells were removed from the top of the filter and cells that migrated were fixed in methanol and stained with DAPI. Migrated cells were manually counted and results were displayed as percentages of invasion relative to scramble.

### Chick chorioallantoic membrane (CAM) assay

To assess *in vivo* tumor formation and angiogenesis, the CAM assay was used, as previously described [33], with some modifications. Briefly, fertilized chicken eggs (Pinto Bar, *n* = 5 for each experiment) were incubated at 37°C;C and 70% humidity. On day 3 of development, after puncturing the air chamber, a hole in a specific region of the eggshell was performed and eggs were sealed with tape and returned to the incubator. On day 10, a small plastic ring was placed on the CAM and 5 × 10^6^ PCa cells (PC3 or LNCaP, control and sh-SMYD3), ressuspended in 20 μL of RPMI or RPMI/F12 medium, were injected in the ring over the CAM. On day 14, the tumor formed was photographed *in ovo* using a stereomicroscope (Olympus S2x16, Olympus, Tokyo, Japan) and, on day 16, chicks were sacrificed at −80°C; C for 10 minutes. The CAM and tumors were fixed with 4% paraformaldehyde and photographed *ex ovo*. Samples (*n* = 10 for each experimental condition) were paraffin-embedded, sectioned, and stained with hematoxylin and eosin (HE) for histological analysis. The total area occupied by tumors was measured using the Cell B software (Olympus) and linear vessel density was assessed by calculating the ratio between the number of vessels and the total length of the membranes.

### Mutagenesis and transient transfection assays

For bacterial expression, *SMYD3* cDNA (Origene Technologies Rockville, MD, USA) was firstly deleted at NHSC or EEL motifs using specific primers (Supplementary Table S2) of the QuikChange II Site-Directed Mutagenesis Kit (Agilent Technologies, Santa Clara, CA, USA). After bacterial transformation, colonies were picked and the plasmids were purified using the Qiagen Plasmid Miniprep Kit (Qiagen, Hilden, Germany). Subsequently, deletions were confirmed by direct sequencing in an ABI PRISM 310 automatic sequencer using Big Dye Terminator Chemistry (Applied Biosystems, Foster City, CA, USA), according to the manufacturer's recommendations. Expression plasmids encoding the mutant *SMYD3* (∆NHSC or ∆EEL) or the wild type *SMYD3* cDNA were transiently transfected into sh-SMYD3 PC3 using LipofectAMINE2000 (Invitrogen), following manufacturer's instructions. Phenotypic assays for cell viability, apoptosis and invasion were performed as described above.

### Identification of putative target genes

To assess whether SMYD3 was implicated in the regulation of selected genes involved in cell cycle, apoptosis, DNA repair, mTOR or MAPK/ERK pathways, a custom array panel (Roche Applied Science, Manheim, Germany) was designed for quantification of expression of those genes. Total RNA was extracted from all cell lines using TRIzol^®^ (Invitrogen) according to manufacturer's instructions and cDNA synthesis was performed using Transcriptor High Fidelity cDNA Synthesis Kit (Roche) according to manufacturer's instructions. Expression levels were determined by real-time PCR in a LightCycler 480 (Roche Diagnostics) and the amounts of mRNA were normalized using *GUSB*, *TFRC* and *18S* as endogenous controls. The comparative Ct method [34] was used to calculate fold-difference in gene expression among groups and only genes with a logarithmized fold change above 1.25 or below −1.25 were further considered.

### Validation of target genes by quantitative RT-PCR (qRT-PCR)

After gene selection, mRNA levels were confirmed in the LNCaP and PC3, both scramble and sh-SMYD3. A total of 1000ng was reverse transcribed using HighCapacity cDNA reverse transcription kit (Applied Biosystems) according to manufacturer's instructions. Expression levels were evaluated using TaqMan^®^ Gene Expression Assays for *AR*, *CCND2*, *C-Met*, *MMP9* and *Nkx2.8* (Applied Biosystems) and GUSB was used as a reference gene. Each plate included multiple non-template controls and all experiments were run in biological and methodological triplicates. Results were always normalized for the result of scramble cell lines established as 1.

### Methylation analysis

DNA extraction from cell lines was performed using a standard technique comprising digestion with proteinase K (20mg/mL) in the presence of 10% SDS at 55°C, followed by phenol-chloroform extraction and precipitation with 100% ethanol. One microgram of DNA was submitted to bisulfite modification using the EZ DNA MethylationGold™ Kit (Zymo Research, Orange, CA) according to manufacturer instructions. Specific CCND2 primers and TaqMan probe were designed using the Methyl Primer Express Software v1.0 (Applied Biosystems). β-actin (ACTB) was used as an internal reference gene to normalize for DNA input. CCND2 methylation levels were calculated after normalization for ACTB and PC-3 and LNCaP scramble samples served as controls to ascertain differential methylation upon SMYD3 silencing.

### Chromatin immunoprecipitation (ChIP) assay

ChIP assay was performed using EZ-Magna ChIP G-Chromatin Immunoprecipitation Kit and the Magna Grip Rack (Millipore), according to the manufacturer's instructions. For each assay, anti-H3K4me3, anti-H3K27me3, anti-H4K20me3, anti-H3, anti-H4 (all from Abcam) and the negative control provided with the kit (normal mouse IgG), were used. DNA quantification was performed in a 7500 Real- Time PCR System (Applied Biosystems), using Power SYBR Green PCR Master Mix (Applied Biosystems). Three gene-specific pairs of primers for each gene promoter were used, in which primers A were located more distant upstream of Transcriptional Start-Site (TSS) and C those that were closer to TSS (Figure 6C and Supplementary Table S2). The relative amount of promoter DNA was calculated for each histone mark over the core histone (H3 or H4) and normalized using Input Percent Method as previously described by our group [35].

### Immunohistochemistry

Immunohistochemistry was performed using the Novolink™ Max Polymer Detection System (Leica Biosystems, Germany]. Sections (3 μm thick) from formalin-fixed and paraffin-embedded tissues of a cohort of 150 primary PCa were used (Supplementary Table S3). Antigen retrieval was accomplished by microwaving the specimens at 800W for 20–30 minutes in EDTA buffer. After cooling the slides, endogenous peroxidase activity was blocked by incubating the sections in hydrogen peroxide in 3% methanol for 30 minutes. Primary monoclonal antibodies for SMYD3 (#61407, Active Motif, Carlsbad, CA, USA) and Cyclin D2 (clone D52F9, Cell Signaling, Danvers, MA, USA) were used in 1:300 and 1:200 dilution, respectively, with 1% PBS-BSA and incubated at 4°C;C overnight. Then, 3, 3′-diaminobenzidine (Sigma-Aldrich™, Germany) was used for visualization and hematoxilin for nuclear counterstaining. Finally, after dehydration and diaphanization, slides were mounted with Entellan^®^ (Merck-Millipore, Germany). Seminal vesicle and colon tissue sections showing intense immunoreactivity for SMYD3 and Cyclin D2 were used as positive controls, respectively. The negative control consisted on the omission of the primary antibodies. Slides were observed at the optical microscope and evaluated for SMYD3 and CCND2 immunoexpression by an experienced Uropathologist. Scoring criteria were: 0 – no immunoexpression; +1 – immunoexpression lower than in normal prostate epithelial cells; +2 – immunoexpression similar to normal prostate epithelial cells; +3 – immunoexpression higher than in normal prostate epithelial cells.

### Statistical analysis

The Shapiro-Wilk's W test allowed for the examination of the appropriateness of a normal distribution assumption for each of the parameters (data not shown). For *in vitro* and *in vivo* assays, comparison between two groups was performed using the Mann-Whitney *U*-test. Differences in immunoexpression frequencies, as well as associations with clinicopathological parameters, were assessed using the Chi-square test. All statistical tests were 2-sided. Statistical analysis was carried out using Graph Pad Prism version 5. Significance level was set at *p* < 0.05.

## SUPPLEMENTARY FIGURES AND TABLES


