# Supplementary material for: SMYD3 contributes to a more aggressive phenotype of prostate cancer and targets Cyclin D2 through H4K20me3
Source: Oncotarget. 2015 Apr 25;6(15):13644–57. doi: 10.18632/oncotarget.3767 (PMC4537039; doi:10.18632/oncotarget.3767)
Supplement: Supplementary file 1 [file oncotarget-06-13644-s001.pdf]

SUPPLEMENTARY FIGURES AND TABLES

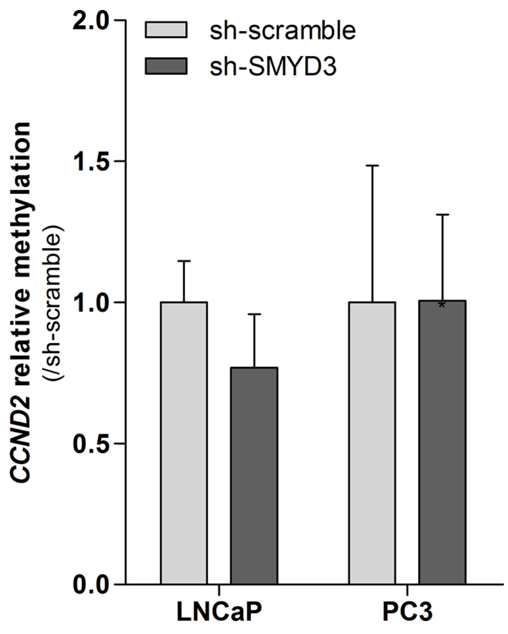

Supplementary Figure S1: Methylation levels of CCND2 by real-time RT-PCR in both scramble and sh-SMYD3 LNCaP and PC3 cell lines.

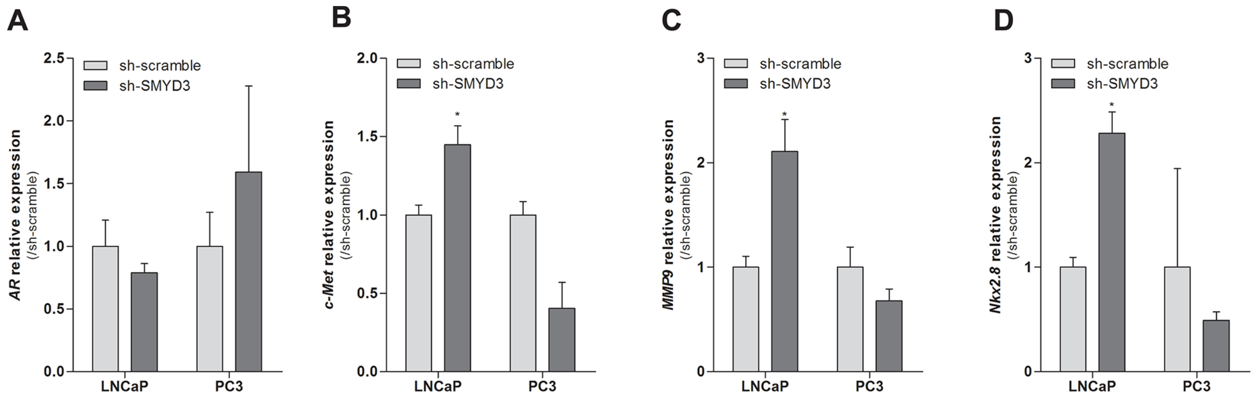

Supplementary Figure S2: Expression levels of *AR* A. *C-Met* B., *MMP9* C. and Nkx2.8 D. by real-time RT-PCR in both scramble and sh-SMYD3 LNCaP and PC3 cell lines. *$p < 0.05$ (Mann-Whitney *U*-test).

Supplementary Table S1: List of differentially expressed genes (fold change above 1.25 or below –1.25) in at least one of the cell lines

	Fold Variation	
**Gene**	**LNCaP**	**PC3**
CCND2	**3.06**	**1.47**
RAD50	0.76	**−1.38**
BBC3	**1.41**	−0.93
CDKN1A	0.49	**1.29**
RAD51	−0.42	**1.34**

Supplementary Table S2: List of primers used in (A) Site-Directed Mutagenesis for deletion of EEL and NHSC domains of SMYD3, (B) direct sequencing of SMYD3 and (C) DNA quantification of several regions of CCND2 promoter in Chromatin Immunoprecipitation

**(A) Site-Directed Mutagenesis**	
Primers EEL	F: CGAGACATCGAGGTGGGAACCATCTGCTACCT
	R: AGGTAGCAGATGGTTCCCACCTCGATGTCTCG
Primers NHSC	F: CCAGTATCTCTTTGCTCGACCCCAACTGTTCGAT
	R: ATCGAACAGTTGGGGTCGAGCAAAGAGATACTGG
**(B) Direct Sequencing of *SMYD3***	
Primers	F: GCCTCAGGCAACTCGTAATG
	R: GAACAAGGCTTCCTCCAACA
**(C) Chromatin Immunoprecipitation for *CCND2***	
Primers A	F: CCAAACTCTTCCTCACCCTTT
	R: CGTACACTAGGTTCCCTGCAA
Primers B	F: GGAAGGGGTGGTGGTGTTT
	R: CCCTGCATCTGCTGACAAG
Primers C	F: GGTTTCTGCTCGAGGATCAC
	R: GTTTCGAAAGCCCCGATTA

F: Forward; R: Reverse

Supplementary Table S3: Clinical and pathological characteristics of the prostate cancer patients' population assessed for SMYD3 and Cyclin D2 immunoexpression

	PCa
**Number of patients, *n***	150
**Age (years), median (range)**	64.5 (49–75)
**PSA levels (ng/mL), median (range)**	8.3 (2.9–23.0)
**Pathological stage, *n* (%)**	
pT2	91 (60.7)
pT3a	48 (32.0)
pT3b	11 (7.3)
**Gleason Score, *n* (%)**	
< 7	58 (38.7)
≥7	92 (61.3)

PCa, prostate cancer
